# Exploiting optical degrees of freedom for information multiplexing in diffractive neural networks

**DOI:** 10.1038/s41377-022-00903-8

**Published:** 2022-07-06

**Authors:** Chao Zuo, Qian Chen

**Affiliations:** 1 Smart Computational Imaging Laboratory (SCILab), School of Electronic and Optical Engineering, Nanjing University of Science and Technology, 210094 Nanjing, Jiangsu Province China; 2 Smart Computational Imaging Research Institute (SCIRI) of Nanjing University of Science and Technology, 210019 Nanjing, Jiangsu Province China; 3 Jiangsu Key Laboratory of Spectral Imaging & Intelligent Sense, 210094 Nanjing, Jiangsu Province China

**Keywords:** Optics and photonics, Optical physics

## Abstract

Exploiting internal degrees of freedom of light, such as polarization, provides efficient ways to scale the capacity of optical diffractive computing, which may ultimately lead to high-throughput, multifunctional all-optical diffractive processors that can execute a diverse range of tasks in parallel.

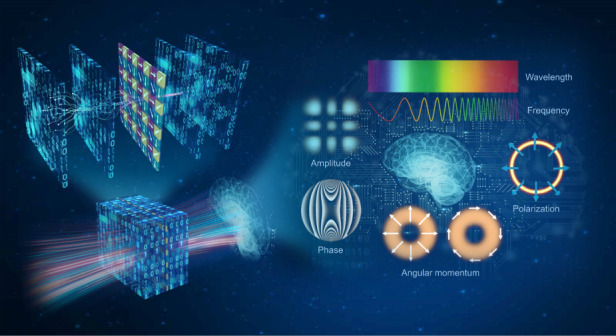

In the last decades, artificial intelligence (AI) technologies, especially artificial neural networks (ANNs), have led to a revolution in a range of applications, including autonomous driving, remote sensing, medical diagnosis, natural language processing, and the Internet of Things. However, the rapid progress of AI and the increasing scale of ANNs are actually accompanied with a tremendous amount of computational resources and energy costs^1^. The main reason behind this is that the dominant computational algorithm for ANNs consists of a large number of matrix-vector multiplications, which are typically the most computationally-intensive operations with the computing cost scales as the square of the input dimension^2^. Optical neural networks (ONNs) built using optical matrix-vector multipliers are promising candidates for next-generation neuromorphic computation, because they offer a potential solution to the energy consumption problem faced by their electrical counterparts^3^. In addition, the constituent scalar multiplication operations can be performed in parallel completely in the optical domain, at the speed of light, and with zero energy consumption in principle^4^.

Optical computing, or more specifically ONNs, where people seek to perform neuromorphic computation with optics, is, in fact, not a new idea. In 1987, Mostafa and Psaltis^5^, for the first time, focused on the need and practical implementation of optical neural computers. Taking inspiration from the distributed topology of the brain, they created a physical implementation of neural networks by arranging optical components in the way neurons are arranged in the human brain. Since then, research in optical neuromorphic computing has flourished, spanning decades of development efforts on various novel optical implementations of neural networks^6^. But until recently experimental implementations of large-scale, highly parallel, high-speed, and trainable ONNs have been made with the breakthroughs in deep learning, optoelectronics, and photonic material engineering, leading to a resurgence of interest in this area.

ONNs are usually built based on an optical architecture that is mathematically described as an input-output function, i.e., a scattering matrix relating the input to the output electric field. And this naturally implements a matrix-vector multiplication, which can be realized by a diverse set of optical architectures, including integrated silicon photonic neuromorphic circuits^7^, fiber-optic sensor arrays^8^, and convolutional networks through diffractive optics^9–11^. Introduced by Ozcan Research Group at the University of California, Los Angeles (UCLA), ONNs formed through the integration of successive spatially engineered transmissive diffractive layers, i.e., diffractive neural networks, have been demonstrated to enable both statistical inference and optical information processing, such as image classification^9^, single-pixel image reconstruction^12^, quantitative phase imaging^13^, and imaging through random diffusers^14^.

The diffractive neural network has its roots in Fourier optics, wherein a simple positive lens applies a physical two-dimensional Fourier transform to the wave field, and the prevalent wave propagation is described by Kirchhoff’s diffraction integral that amounts to a convolution of the field with the impulse response of free space. These operations provide basic building blocks of convolutional neural networks (CNNs), making diffractive neural networks well-suited for most vision computing applications. By leveraging the light-matter interaction as an implementation of element-wise multiplication, the “pixels” on the diffractive surfaces embody the “neurons” on the network layers, which are interconnected by the physics of optical diffraction. As an analogy to standard neural networks, the complex-valued transmission coefficient (including amplitude and phase) of each pixel is a learnable network parameter, which is iteratively optimized based on error back-propagation algorithms, using standard deep learning tools implemented in a computer. After this training stage, the resulting transmissive layers are fabricated with 3D printing or lithography to construct a task-specific physical network that computes based on the diffraction of the light passing through these trained diffractive layers.

Though most of the current diffractive neural networks are constructed based on linear optical materials, “deep” diffractive neural networks show evident “depth” abvantages: an increase in the number of diffractive layers and neurons improves its statistical inference accuracy and information processing capability^9,15^. More specifically, adding more trainable diffractive layers into a given network increases the dimensionality of the solution space that can be all-optically processed by the network. It has been recently demonstrated that a diffractive neural network can be trained to perform an arbitrary complex-valued linear transformation between its input and output fields with negligible error, provided that the total number of engineered pixels in the network is sufficient^16^. In a more general sense, a diffractive neural network can be regarded as a special, task-specific optical system, which performs specific computational tasks with the use of light information carriers. The object field can be viewed as a source of information flow characterized by various fundamental properties, which can all be ingeniously manipulated to extend the information processing capacity of diffractive networks.

In a recent issue of *Light: Science & Applications*, the UCLA group introduced polarization division multiplexing (PDM), a long-established technique of enhancing the transmission capacity in telecommunications, to all-optically perform multiple, arbitrarily-selected linear transformations through a single diffractive network^17^ (Fig. 1a). Instead of using birefringent, anisotropic, or polarization-sensitive materials for trainable diffractive layers, their polarization-multiplexed diffractive networks are still built based on standard (isotropic) diffractive surfaces where the trainable coefficients are independent of the polarization state of the input light. To gain additional sensitity to different polarization states and polarization mode diversity, a non-trainable, pre-selected linear polarizer array (at 0°, 45°, 90°, and 135°) is inserted within the trainable diffractive surfaces, and different target linear transformations are uniquely assigned to different combinations of input and output polarization states. They demonstrated that a single well-trained polarization-multiplexed diffractive network could successfully perform multiple (2 or 4) arbitrarily-selected linear transformations, which had not yet been implemented by using metasurfaces or metamaterials-based designs^11,17^. Such a polarization-multiplexed diffractive computing framework is poised to be used to build all-optical, passive processors that can execute multiple inference and optical information processing tasks in parallel.

**Fig. 1:**
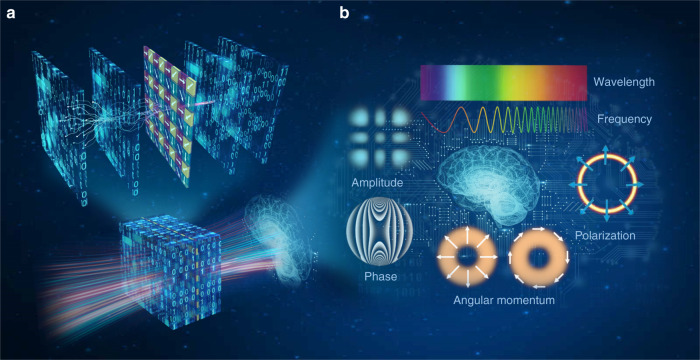
Information multiplexing in diffractive neural networks. (**a**) Polarization-multiplexed diffractive neural networks utilizing a series of structured diffractive surfaces and a simple polarizer array. By enabling the trainable diffractive layers to communicate with the polarization elements embedded in the diffractive volume, a single network can create multiple computing channels that can be accessed using specific combinations of input and output polarization states. (**b**) Exploiting the internal degrees of freedom of light provide new possibilities for information multiplexing to enhance the performance and capacity of optical diffractive networks

Harnessing the intrinsic high-dimensionality of light brings new insights into the diffractive neural network design by providing additional *degrees of freedom* to both optical signals and systems. The concept of degrees of freedom was first introduced in optics by Laue^18^ in 1914 as the decisive property in determining the information capacity of optical signals and systems, even before Shannon’s information theory for communication systems was established^19^. According to Laue^18^, and later Gabor^20^, Francia^21^, and Lukosz^22,23^, the number of degrees of freedom in optics is most often understood to be the number of independent parameters needed to represent an optical signal or system, which is closely related to the number of independent communication channels available for the information transfer in the field of electrical communication. However, unlike communication systems, an optical system transmits many kinds of information, which can be divided into two groups: (1) “*dimensional*” information which is related to spatial intervals by coordinates x, y, and z, as well as to temporal intervals t; (2) “*internal*” information which is related to physical properties of light, including amplitude, phase, wavelength, polarization, coherence, and angular momentum. The total number of degrees of freedom can be expressed through the product of freedom degree numbers related to all these different kinds of information. Though optical systems are often expected to transmit as much information as possible, the number of degrees of freedom is a fundamental invariant of an optical system, as noted by Gabor^20^ and Lukosz^22^. Within this limit, it is possible to increase the degrees of freedom for one kind of information at the expense of that of another kind^22,23^.

The concept of degrees of freedom can be straightforwardly extended to the diffractive neural network, as a special kind of optical signal processing system. For example, the information content of the input or output signal, which is often an image formed through a pupil of finite size, can be quantified by the definite number of resolvable regions in which the signals can be independent (defined as *N*_*i*_ and *N*_*j*_ for the input or output signal in ref. ^15^), taking both the diffraction limit and sampling theorem into account^21,22^. Diffractive neural networks manipulate light by reshaping the spatial profile of an input beam into a desired output beam. If the diffractive neural network is designed to perform arbitrary linear transformations from the input beam to the output beam, as demonstrated in refs. ^15,16^, the entire optical system can be described by a single *N*_*i*_×*N*_*j*_ matrix, mapping *N*_*i*_ input degrees of freedom to *N*_*j*_ output degrees of freedom. In optical communications, the concept of “modes” or “eigenfunctions”, is commonly used to provide an “economical” description of degrees of freedom of the optical signal, reducing complicated wave functions to a small number of mode amplitudes, as in propagating fiber modes and ideal laser beams^24^. In such a sense, the linear transformation function realized by the diffraction neural network is similar to that of an optical mode converter^25^. In contrast, diffractive neural networks can be built in a “deep” manner, consisting of several diffractive surfaces containing a large number of trainable neurons. Such a multi-layer design presents additional spatial optical degrees of freedom, significantly enhancing the information capacities and processing capability of the network compared with a single diffractive layer, as demonstrated by the UCLA group^15–17^. In particular, any linear transformation matrix from *N*_*i*_ to spatial degrees of freedom has *N*_*i*_ *×* *N*_*j*_ free parameters. When the degrees of freedom of the diffractive neural network, i.e., the number of controllable parameters, is no less than *N*_*i*_×*N*_*j*_, the network has in theory the capability to perform arbitrary linear transformations between the input and output signals perfectly. In their recent work^17^, two additional degrees of freedom of polarization are introduced to the input signal for simultaneously carrying the different information through the network. The two orthogonal polarization states carried by the beam present an attractive avenue to enhance the maximum information capacity of the diffractive neural network by a factor of *N*_*p*_ (from *N*_*i*_ *×* *N*_*j*_ to *N*_*p*_ *×* *N*_*i*_ *×* *N*_*j*_), where *N*_*p*_ is the number of unique linear transformations assigned to different input/output states of polarization combinations. It should be mentioned that the use of polarization freedom as high-dimensional information carriers has been reported by Lohmann et al.^26^ for optical super-resolution imaging and Chen et al.^27^ in optical data communications.

The study of the UCLA group published in *Light Science & Application* is part of a larger movement to scale the capacity of optical diffractive computing by exploiting the *internal* degrees of freedom of light, such as polarization, spectrum, coherence, and orbital angular momentum, in addition to the *spatial* degrees of freedom (Fig. 1b). With such a multidimensional multi-link upgrade, diffractive neural networks can transmit optical signals over more independent channels, which could lead to all-optical multiplexed diffractive processors that can execute multiple tasks in parallel. Another benefit of polarization multiplexing is that the effective bandwidth can be reduced to the half of that of single-polarization transmission. That makes a high-dimensional diffractive neural network possible by using lower numerical-aperture optics, which has been proved to be extremely important for reducing the physical size of diffractive neural networks and relaxing the stringent requirements on the interlayer distances^15,16^. Finally, in most current diffractive network designs, the input field is assumed to be monochromatic, spatially coherent, and forward-propagating. A variety of computational imaging techniques that exploit partial coherence and evanescent waves for improving imaging performance (especially spatial resolution) prompted us to consider the possibility of their adaptation to diffraction neural networks. We believe that significant progress in developing high-performance optical diffractive computing schemes could be made if it became common practice to consider explicitly the internal degrees of freedom of light as the physical source of information gain.
